# Response of Syngeneic Murine Lymphomata to Immunotherapy in Relation to the Antigencity of the Tumour

**DOI:** 10.1038/bjc.1972.24

**Published:** 1972-06

**Authors:** Iris Parr

## Abstract

The responses of four murine lymphomata, transplanted in their syngeneic hosts and differing widely in their biological properties such as tendency to metastasize and “strength” of tumour specific antigen, to immunotherapy, were investigated. Following the injection of a known number of tumour cells, the mice were treated either by administration of irradiated tumour cells, living BCG or both. In general, the response to BCG alone was small even in the most responsive tumours, irradiated cells were more effective and the best results were obtained by a combination of the two procedures. “Cures” were only obtained with the most antigenic of the tumours tested and then only when the live tumour cells were inoculated intraperitoneally. The effect of these treatments on the rate of growth of tumour when the cells were given subcutaneously was small but the rate at which metastatic spread occurred from the s.c. site was slowed and for one of these tumours this was quite marked.


Br. J. Cancer (1972) 26, 174

### RESPONSE OF SYNGENEIC MURINE LYMPHOMATA TO IMMUNOTHERAPY IN RELATION TO THE ANTIGENICITY OF THE TUMOUR

IRIS PARR

*Chester Beatty Research Institute, Clifton Avenue, Belmont, Sutton, Surrey*

Received for publication November 1971

**Summary.—The responses of four murine lymphomata, transplanted in their syngeneic hosts and differing widely in their biological properties such as tendency to metastasize and " strength " of tumour specific antigen, to immunotherapy, were investigated. Following the injection of a known number of tumour cells, the mice were treated either by administration of irradiated tumour cells, living BCG or both. In general, the response to BCG alone was small even in the most responsive tumours, irradiated cells were more effective and the best results were obtained by a combination of the two procedures. " Cures " were only obtained with the most antigenic of the tumours tested and then only when the live tumour cells were inoculated intraperitoneally. The effect of these treatments on the rate of growth of tumour when the cells were given subcutaneously was small but the rate at which metastatic spread occurred from the s.c. site was slowed and for one of these tumours this was quite marked.**

PROTECTION of animals against a syngeneic tumour graft by prior immunization with cells from the same tumour, that have been rendered incapable of growth by exposure to x-rays, constitutes evidence for the presence of tumour specific antigen of the transplantation type. The growth of some syngeneic tumours is retarded or prevented in animals that have, prior to graft, received treatments that stimulated the reticuloendothelial system, such as the administration of Bacillus Calmett–Guerin (BCG) (Old *et al.*, 1961; Weiss, Bonhag and De Orme, 1961), or *Corynebacterium parvum* (Woodruff and Boak, 1966).

The administration of BCG or *C. parvum*, besides protecting prophylactically, will slow the growth of some established tumours (Mathé, Pouillart and Lapeyraque, 1969; Woodruff and Boak, 1966). A very striking effect (Currie and Bagshaw, 1970) was obtained by administering *C. parvum* after the tumour size had been reduced by chemotherapy. With the L1210 murine lymphoma Mathé observed that a combination of BCG with irradiated tumour cells was more effective than either treatment alone.

Haddow and Alexander (1964) found in rats that injection of irradiated autologous tumour cells (obtained by biopsy from autochthonous chemically induced sarcoma) slowed the growth of the primary tumour but only if the amount of tumour present was small. This observation was also made by Mathé (Mathé, 1968; Mathé *et al.*, 1969) who treated mice carrying a subcutaneous graft of L1210 tumour cells with irradiated syngeneic tumour cells. A possible mechanism was suggested by the finding in rats (Alexander *et al.*, 1969) that the function of the node draining the primary tumour is impaired, and that by stimulating uninvolved nodes with irradiated tumour cells the cell-mediated host response was increased.

In this paper we report experiments to study the effect of giving irradiated cells, BCG and the two procedures combined, on the growth in syngeneic hosts of 4 murine lymphomata which differ widely

TABLE I.—*Summary of Properties of Murine Lymphomata Described in Test*

| Tumour* | Mouse of origin | Method of induction (date) | Immunogenicity† | Capacity‡ to metastasize |
|---|---|---|---|---|
| L5178Y | DBA/2 | Methylcholanthrene 1961 | + + + + | + |
| L5178Y-M | DBA/2 | From L5178Y 1968 | + | + + + + |
| TLC5 | CBA | Methylcholanthrene 1968 | + + | + + + + |
| TLX9 | C57Bl | Whole-body x-irradiation 1967 | + | + + + |

* In the case of all 4 tumours injection of 10 i.p. cells gave 100% mortality.
† Measured in terms of immunization against an i.p. live cell challenge.
‡ From a s.c. site of implant.

in their biological properties, such as the strength of their tumour specific antigens and their capacity to metastasize.

### METHODS

The mice were reared under specific pathogen-free conditions, kept caged in groups of 5 and fed food and water *ad libitum.*

The murine lymphomata used in these studies (Table I) were induced chemically or by x-irradiation and have been adapted to grow as ascitic tumours in the peritoneal cavity. They were maintained by weekly serial passage of washed, counted tumour cells. At 4–5 monthly intervals the tumours were re-established from stock cells from a tumour bank maintained in liquid nitrogen.

Tumour cell suspensions were irradiated with 250 kpV x-rays. To ensure that the cells were oxygenated the cell suspensions were diluted to $2 \times 10^6$ cells/ml, or in some cases $10^7$ cells/ml, with ice-cold TC 199 medium just before irradiation. A dose of 3000 rad given under these conditions was sufficient to stop an inoculum of $10^8$ cells from growing when injected. Dried BCG vaccine (in the form for percutaneous injection) was kindly supplied by Glaxo.

*Assay of immunogenicity of tumours.*—Mice were immunized intraperitoneally (i.p.) with measured doses of irradiated syngeneic tumour cells and subsequently challenged 7 days later with serial dilutions of viable tumour cells. The degree of tumour immunity achieved was measured in terms of the number of mice free of tumour 6 weeks after tumour challenge.

*Assessment of metastasizing properties of the tumours.*—This was made in terms of the survival time of mice given subcutaneous (s.c.) implants of tumour. Cells metastasized mainly to the liver and spleen.

FIG. 1.—Mortality curves for the 4 lymphomata when $10^5$ cells are injected s.c. Ten animals per group. I, TLC5; II, L5178Y-M; III, TLX9; IV, L5178Y-(s.c.); V, L5178Y-(i.d.).

### RESULTS

### 1. *Properties of the tumours*

Table I gives brief details of the 4 lymphomata used in this study, viz. method and date of induction, immunogenicity and capacity to metastasize. The experimental basis for the summary is given in Tables II and III. With regard to capacity to metastasize from the site of the primary inoculum the lymphomata vary greatly (Fig. 1). Mice carrying TLC5 or L5178Y-M as a subcutaneous graft die with widespread metastases to liver and spleen when the primary tumour mass is 3–4 mm in diameter, while L5178Y-original line tumour cells will grow to form a substantial subcutaneous tumour approximately 20 × 20 mm in 4–5 weeks. At this time there is microscopic infiltration of the liver in some of the animals but in others the liver and spleen are only slightly heavier than normal values. Intradermal transplants of L5178Y metastasize rather less frequently from the primary site; the few deaths recorded (Fig. 1) during the 5–6 weeks the animals were kept alive were the result of tumours invading the body wall and entering the body cavity.

An increased facility to form metastases is paralleled by a decisive fall in immunogenicity (Table I). This is particularly marked in the case of the 2 lines of L5178Y, where one is immunogenic and non-metastasizing, L5178Y, while the other L5178Y-M is metastasizing and but weakly immunogenic.

Tables II and III give details of the antigenic properties of the four lymphomata studied. Although in general the L5178Y tumour possessed by far the strongest tumour specific antigens the actual level of immunogenicity recorded for this lymphoma was dependent upon the site of injection of the live tumour cell challenge, *e.g.* intraperitoneal or subcutaneous. Thus DBA/2 mice rendered immune to an intraperitoneal challenge of $10^6$ L5178Y cells were not protected against the same dose of cells given s.c. Immunotherapy techniques were therefore investigated on both intraperitoneal and subcutaneously growing tumours. In the case of the less immunogenic tumours, *e.g.* TLC5, the degree of protection afforded by immunization was much less for both s.c. and i.p. challenges and here the differences between the two sites were not so apparent.

TABLE II.—*Immunogenicity of 4 Murine Lymphomata Measured Against an Intraperitoneal Challenge*

| Tumour | No. of immunizations* | No. of irradiated cells/immunization | Route of immunization | Titration with grade cell doses† Survivors — Total no. injected with tumour cells: $10^2$ | $10^3$ | $10^5$ | $10^6$ | $10^7$ | $10^8$ |
|---|---|---|---|---|---|---|---|---|---|
| L5178Y | 1 | $5 \times 10^6$ | i.p. | 10/10 | 10/10 | 10/10 | 9/10 | 0/10 | |
| L5178Y-M | 1 | $10 \times 10^6$ | multi-site‡ | 4/5 | 1/5 | 0/5 | | | |
| TLC5 | 1 | $25 \times 10^6$ | multi-site | 4/10 | 1/10 | | | | |
| TLX9 | 1 | $100 \times 10^6$ | multi-site | 0/10 | | | | | |
| L5178Y | 2 | $5 \times 10^6$; $5 \times 10^6$ | i.p. | | | | 10/10 | 6/10 | |
| | 3 | $5 \times 10^6$; $5 \times 10^6$; $5 \times 10^6$ | | | | | 10/10 | 10/10 | 6/10 |
| L5178Y-M | 2 | $10 \times 10^6$; $10 \times 10^6$ | multi-site | 3/5 | 3/5 | 0/5 | | | |
| TLC5 | 2 | $10 \times 10^6$; $20 \times 10^6$ | multi-site | N.T. | 10/10 | 8/10 | 10/10 | N.T | |
| TLX9 | 2 | $10 \times 10^6$; $20 \times 10^6$ | multi-site | 3/10 | 2/10 | N.T. | | | |
| | 3 | $10 \times 10^6$; $20 \times 10^6$ $20 \times 10^6$ | multi-site | 8/10 | 3/10 | 0/10 | | | |

*Separated by 7 days.
† Given 7 days after last immunization.
‡ Irradiated cells divided equally between 4 s.c. sites and i.p.—1/5 total cells/site.
*N.B.*: For all the tumours listed 10 cells i.p. gave 100% mortality.

TABLE III.—*Immunogenicity of 2 Murine Lymphomata Measured Against a Subcutaneous Challenge*

| Tumour | No. of immunizations | No. of irradiated cells/immunization | Route of immunization | Titration with graded cell doses Survivors* / Total no. injected with tumour cells s.c. $10^4$ | $10^5$ | $10^6$ | $10^7$ |
|---|---|---|---|---|---|---|---|
| L5178Y | 1 | $5\times10^6$ | i.p. | 2/5 | 1/5 | 0/5 | NT |
| | 1 | $40\times10^6$ | multi-site | | 3/5 | 2/5 | |
| | 2 | $5\times10^6$; $5\times10^6$ | i.p. | 5/5 | 4/5 | 2/5 | 1/5 |
| | 3 | $5\times10^6$; $5\times10^6$ $5\times10^6$ | i.p. | NT | 5/5 | 4/5 | 3/5 |
| TLC5 | 1 | $25\times10^6$ | multi-site | 2/5 | 0/5 | | |
| | 2 | $10\times10^6$; $25\times10^6$ | multi-site | 5/5 | 3/5 | 0/5 | |

* Animals free of tumour 60 days after implant.
*N.B.:* For L5178Y $10^5$ cells s.c. gave 100% mortality; for TLC5 $10^2$ cells s.c. gave 100% mortality.

2. *Effect of immunotherapy on the growth of intraperitoneal tumour*

*L5178Y lymphoma - non - metastasizing line.*—Sixty mice (DBA/2) received $10^3$ L5178Y lymphoma cells i.p. and were then divided into 4 groups. The first was the control group and received no further treatment. The second was injected i.p. with 40–50 × $10^6$ irradiated tumour cells 24 hours after the live lymphoma cells. The third group received one i.p. injection of BCG (equivalent to 0·6 mg w/w of bacteria) 24 hours after tumour challenge, while the final group was injected i.p. with both irradiated tumour cells and BCG (given 3 hours after the irradiated cells) 24 hours after tumour challenge.

BCG alone had little effect on the rate at which this tumour killed the animals.

FIG. 2.—Immunotherapy of L5178Y lymphoma growing i.p. $10^3$ L5178Y cells injected i.p. on day 0. All treatments (except for one group) given 24 hours after tumour cells. ●, Death of mouse; ○→, survivors free of tumour.

FIG. 3.—Immunotherapy of L5178Y growing i.p. Effect of mixing BCG and irradiated cells together before injection. Treatments given 24 hours after tumour cells. ●, Death of mouse; ○→, survivors free of tumour.

Irradiated tumour cells alone had a decisive therapeutic effect and administration of both BCG and irradiated cells protected 66 % of the mice (Fig. 2). When immunotherapy was delayed until 3 days after tumour transplant, very little protection was afforded to the mice (Fig. 2); when the interval was 7 days the treated animals died at the same time as the controls. If BCG and irradiated cells were mixed together prior to injection, the protective effects of both treatments were cancelled (Fig. 3).

The controls died with ascites and widespread tumour masses in the peritoneal cavity. There was little tumour spread to the liver or spleen. Those treated animals which died at the same time as the controls presented a similar picture at post-mortem; whereas those animals which died 10–20 days after the controls did not have ascites and few animals had tumour masses in the peritoneal cavity. The main post-mortem feature in these animals was an enlarged liver and in some cases an enlarged spleen, which on histological examination proved to be widely infiltrated with lymphoma cells.

Various alterations in the experimental routine such as increase in the number of irradiated cells (up to $100 \times 10^6$), repeated doses of irradiated cells and changes in route of administration, did not appear to have much effect on the number of " cures " obtained (Table IV).

TABLE IV.—*Immunotherapy with Irradiated Cells against i.p. Tumour. Effect of Changing Numbers of Cells Given and Route of Injection*

| Immunotherapy: Route of injection | Immunotherapy: No. of irradiated cells | Survivors*/Total no. of animals implanted with $10^3$ i.p. L5178Y cells |
|---|---|---|
| i.p. | $40 \times 10^6$ | 2/5 |
| i.p. | $100 \times 10^6$ | 2/5 |
| s.c. | $40 \times 10^6$ | 2/5 |
| i.p. | $40 \times 10^6$ (repeated $\times 2$ per week) | 2/5 |
| | None (controls) | 0/5 |

* Mice free of tumour 60 days after implant.

Survivors of the experiment just described were killed 4–6 months after

FIG. 4.—(a) Immunotherapy of TLC5 lymphoma growing i.p. $10^2$ TLC5 lymphoma cells were injected i.p. day 0. Treatment given 4 hours after tumour cells. (b) Immunotherapy of TLX9 lymphoma growing i.p. $10^2$ TLX9 cells injected i.p. on day 0. Treatment given 24 hours after tumour cells. (c) Immunotherapy of L5178Y-M growing i.p. Treatment given 24 hours after tumour cells ($10^3$) i.p. ●, Death of mouse; ○→, survivors free of tumour.

tumour challenge and tissues (liver, spleen and lymph nodes) were taken from a representative number and examined histologically for tumour cell infiltration. None was found.

*L5178Y-M lymphoma.*—The experiments followed a similar pattern to those described in the previous section except that groups treated with BCG only were omitted. Fig. 4 illustrates the complete lack of response of this tumour to immunotherapy.

*TLC5 and TLX9 lymphomata.*—Again the design of experiments was the same as that described for the L5178Y line, except that the aliquots of irradiated cells were injected at 4 s.c. sites and i.p. 2–4 hours after the live tumour cell challenge in the case of TLC5. Slight prolongation of life was observed in some of the treated mice but in terms of surviving animals, immunotherapy had virtually no effect.

### 3. *Effect of immunotherapy on the growth of subcutaneous tumour*

*L5178Y lymphoma.*—In preliminary experiments using small tumour cell inocula of $10^3$ and $10^4$, immunotherapy, if it had any effect at all, appeared to accelerate the rate of growth of the s.c. tumours. However, using this route of injection 100% " take " of the tumour graft was not achieved with inocula less than $10^5$. For the main series of experiments 40 mice were injected s.c. in the flank with $10^5$ cells (in 0·1 vol.), then divided into 4 groups. Ten controls received no further treatment. Twenty-four hours later 10 mice were injected i.p. with $40 \times 10^6$ irradiated cells, a further 10 received BCG, and the final group was given irradiated cells $40 \times 10^6$ and BCG with a 3–4 hour interval between the 2 treatments.

The rate of growth of the primary tumour was not changed by any of these treatments. On post-mortem examination of the animals (5 weeks after tumour cell implant) macroscopic infiltration of the liver was present in more of the animals in the control and BCG-treated groups than in the other 2 groups (Table V).

TABLE V.—*Comparison of the Number of Animals with Liver Metastases 5 Weeks after $10^5$ L5178Y Lymphoma Cells Transplanted s.c. and Followed by " Immunotherapy "*

| Type of treatment | No. of animals with macroscopic liver infiltration / Total no. of mice injected with $10^5$ s.c. |
|---|---|
| None | 6/10 |
| * BCG 24 hours after tumour | 8/10 |
| * Irradiated cells $40 \times 10^6$ 24 hours after tumour | 3/10 |
| * Irradiated cells $40 \times 10$ and BCG 24 hours after tumour | 2/10 |

* Given i.p.

*L5178Y-M lymphoma.*—Experiments of similar design to those described for the original line of L5178Y failed to demonstrate any effect whatsoever on the rate of growth of the s.c. tumour. The animals did not survive long enough for the tumour to ulcerate but died approximately 21 days after transplant of $10^4$ cells, with widespread metastases in the liver and spleen.

*TLX9.*—Here again, although quite sizeable tumours were formed, the treatment had no effect either on the size of the tumour or on the times of survival.

*TLC5.*—An inoculum of $10^4$ tumour cells was given s.c. to 80 animals which were divided into 4 groups of 20 animals each. One group had no further treatment, one group received irradiated cells ($40 \times 10^6$/mouse), one group received BCG, and the final group both irradiated cells ($40 \times 10^6$/mouse) and BCG. Treatment was given 4 hours after tumour cells. All 20 of the control animals died between 12 and 15 days. Similarly, BCG-treated animals died between day 12 and day 14 after tumour cell implant. Animals in these groups had very small subcutaneous tumours but there were widespread metastases in the liver, spleen and lymph nodes. In those mice given irradiated cells or irradiated cells plus BCG, longer periods of

FIG. 5.—Immunotherapy of TLC5 lymphoma growing s.c. $10^4$ TLC5 cells injected s.c. on day 0. Treatment given 2 hours later. ●, Death of mouse.

FIG. 6.—Failure of immunotherapy to influence growth of primary inoculum. Rate of growth of TLC5 lymphoma injected s.c. ($10^4$). . . . . . . Control, –·–·–·–·– BCG, both graphs ceased day 12 because all animals died days 12–14—cf. Fig. 5. ——— Irradiated cells ($40 \times 10^6$) and BCG – – – – – Irradiated cells ($40 \times 10^6$).

survival were found (Fig. 5). Thus, although in effect the tumours in the treated animals grew to a larger size than in the control and BCG groups (Fig. 6), this was a direct result of longer survival periods. These treatments, as in the case of the L5178Y, had no effect on size of subcutaneous tumour but reduced the number of metastases.

4. *The effect of immunotherapy on the growth of intradermal tumour*

*L5178Y lymphoma.*—A group of 40 animals was treated in the same manner as in the experiments described in the previous section except that the tumour cells were injected intradermally. The rate of tumour growth was slowed in the group given BCG. All the animals were killed 4–5 weeks after implantation because of bad ulceration in some of the tumours. Although the average tumour weight of the group treated with BCG was smaller than that of the control group, this difference was not statistically significant. Tumour cell infiltration of the liver was macroscopically observed in 2 of the controls only.

### DISCUSSION

It appears that the primary immune response induced by a tumour graft of L5178Y cells given by the i.p. route is either not strong enough or is not mounted in time to overcome the progressively increasing numbers of ascitic cells. As few as 10 cells can kill 26–28 days after implant. Further stimulation of the immune mechanism by active immunotherapy, specific (irradiated cells) or non-specific (BCG) or a combination of the 2, given 24 hours after live tumour cells greatly increased the average survival time of mice carrying an i.p. tumour, and in certain instances " cures " were achieved. Under these experimental conditions, *i.e.* when the tumour cell challenge with L5178Y is i.p., addition of BCG increased the efficiency of specific immunotherapy (Fig. 2). Immunotherapy was effective only when the tumour cell burden was small—treatment delayed until 3 days after live tumour implant was not successful. The irradiated cells were given i.p. in most experiments but were just as effective by the subcutaneous route. Thus, the inflammatory type of stimulation which could result from the injection of $40 \times 10^6$ irradiated cells into the peritoneal cavity is not essential for the curative effects of this treatment to be manifest.

The apparent failure of immunotherapy to protect fully against an i.p. implant of the metastasizing TLC5 lymphoma could be explained in terms of the lower antigenicity of this tumour (2 immunizations are necessary to protect against a challenge of $10^6$ cells i.p.). Although in terms of survivors the experiments with TLC5 and TLX9 were failures the small degree of protection afforded by specific immunotherapy might encourage the use of these tumours or ones of similar type as models on which might be tested and developed new and better immunotherapy techniques.

Immunotherapy had no effect on the rate of growth of any of the 4 lymphomata when the cells were grafted in a s.c. site, but it reduced the rate of tumour metastases to the liver. This effect was detected in the series of experiments with L5178Y but was more easily demonstrated in the metastasizing lymphoma TLC5 where survival times could be taken as a measure of rate of metastases formation. In neither series of experiments did BCG noticeably increase the efficiency of specific immunotherapy.

The failure of immunotherapy in our experiments to influence the rate of growth of s.c. tumours contrasts with results obtained by Mathé (1968) and Mathé *et al.* (1969) where complete regression of some of the s.c. tumours of L1210 (murine lymphoma) occurred. In other mice the increase in tumour size was halted and held at a plateau level for several weeks. Possibly in this latter group of animals a reduction in the number of metastases to the liver was responsible for prolongation of life, as in the present experiments.

Although the rate of growth of L5178Y as a s.c. tumour was unaffected by immunotherapy, when the cells were placed intradermally, BCG had a small growth-retarding effect. Intradermally placed lymphoma cells grow at the same initial rate, *i.e.* the tumours attain the same size as tumours growing in a s.c. site; but during the second week after transplant, the rate of growth of the intradermal tumours is slowed. This could be the result of an immune reaction on the part of the animal to cells placed in this position. It was not possible to increase the specific element further by treatment with irradiated cells but stimulation of the reticuloendothelial system with BCG slowed tumour growth.

Comparison of the 4 tumours in terms of immunogenicity and metastasizing properties showed that the one that responded most easily to immunotherapy L5178Y, was the least metastasizing and the most immunogenic. The loss of immunogenicity that occurred as the M line of L5178Y evolved was accompanied by an increased ability to metastasize from both i.p. and s.c. sites and total failure to respond to the type of immunotherapy applied. On the other hand, TLC5 which metastasizes just as rapidly from a s.c. site can be influenced by immunotherapy. It is perhaps significant that the immunogenicity of this tumour (measured in terms of resistance of immunized animals to graded challenge dose inocula) is greater than that of L5178Y-M line. The lymphoma TLX9 which metastasizes but not quite so readily as TLC5 and L5178Y-M responded but slightly to immunotherapy and then only when the live cells were given i.p. The antigenicity of this tumour was as low or lower even than that of L5178Y-M line.

I wish to thank Professor P. Alexander and G. Hamilton Fairley for helpful discussion and criticism. I am grateful to Miss E. Wheeler for excellent technical assistance.

This work was supported by grants made to the Chester Beatty Research Institute by the Medical Research Council and the Cancer Research Campaign and by a grant from the Napier Trust.

### REFERENCES

ALEXANDER, P., BENSTED, J., DELORME, E. J., HALL, J. G. & HODGETT, J. (1969) The Cellular Immune Responses to Primary Sarcomata in Rats. II. Abnormal Responses of Nodes Draining the Tumour. *Proc. R. Soc., Series B*, **174**, 237.

CURRIE, G. A. & BAGSHAW, K. D. (1970) Active Immunotherapy with *Corynebacterium parvum* and Chemotherapy in Murine Fibrosarcomas. *Br. med. J.*, i, 541.

HADDOW, A. & ALEXANDER, P. (1964) An Immunological Method of Increasing the Sensitivity of Primary Sarcomas to Local Irradiation with x-rays. *Lancet*, i, 452.

MATHÉ, G. (1968) Immunothérapie Active de la Leucémie L1210 Appliquée après la Greffe Tumorale. *Revue fr. Étud. clin. biol.*, **13**, 881.

MATHÉ, G., POUILLART, P. & LAPEYRAQUE, F. (1969) Active Immunotherapy of L1210 Leukaemia Applied after the Graft of Tumour Cells. *Br. J. Cancer*, **23**, 814.

OLD, L., BENACERRAF, B., CLARKE, D. A., CARSWELL, E. A. & STOCKERT, E. (1961) The Role of the Reticuloendothelial System in the Host Response to Neoplasia. *Cancer Res.*, **21**, 1281.

WEISS, D. W., BONHAG, R. S. & DE ORME, K. B. (1961) Protective Activity of Fractions of Tubercle Bacilli. *Nature, Lond.*, **190**, 889.

WOODRUFF, M. F. A. & BOAK, J. L. (1966) Inhibitory Effect of Injection of *Corynebacterium parvum* on the Growth of Tumour Transplants in Isogenic Hosts. *Br. J. Cancer*, **20**, 345.


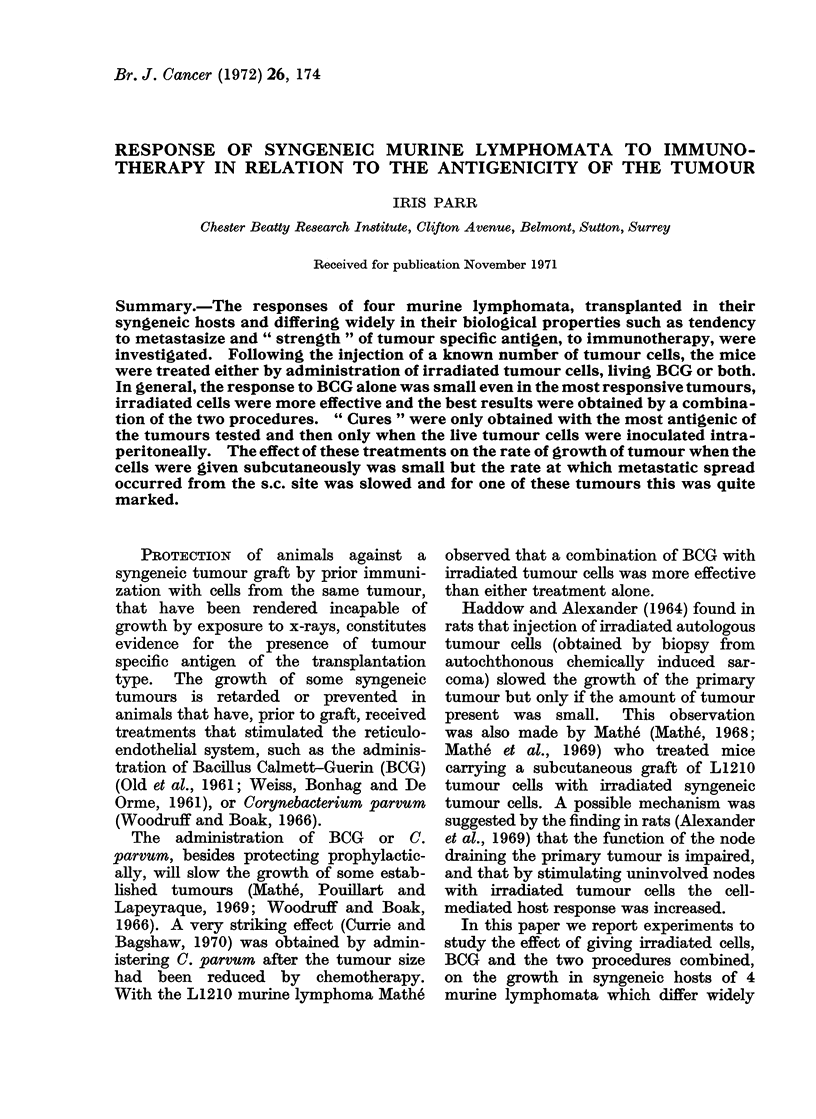

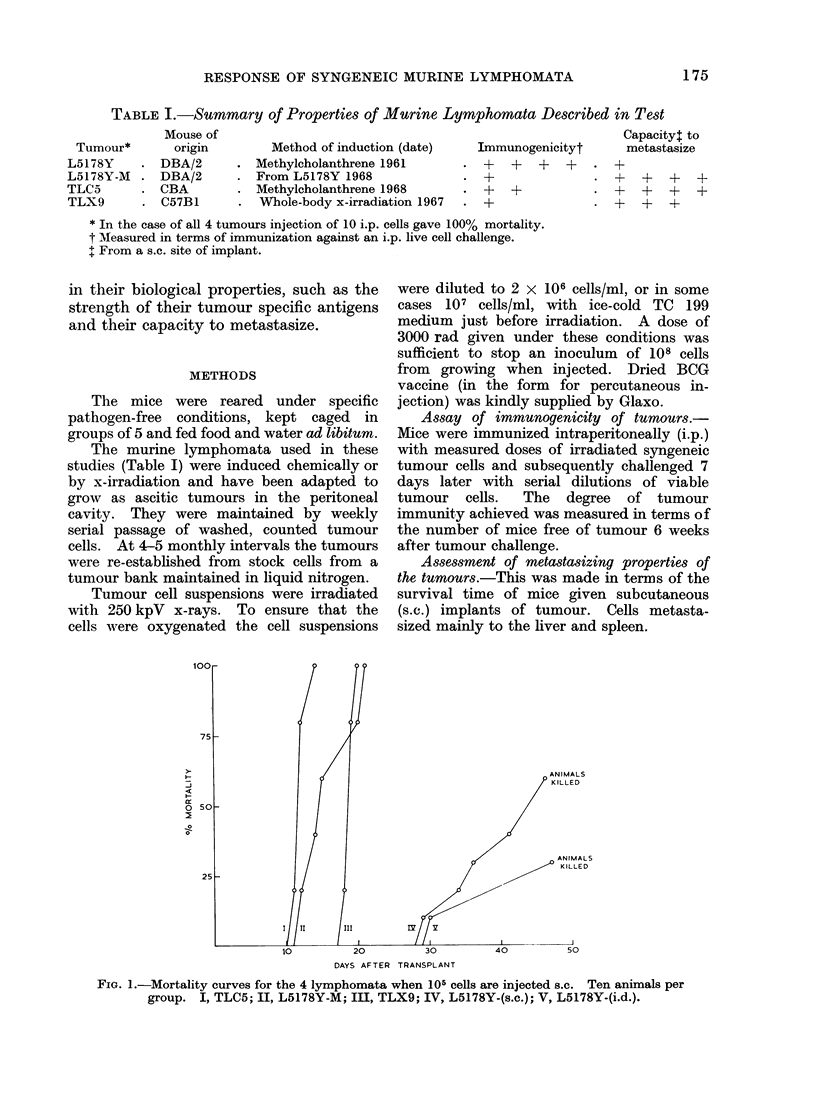

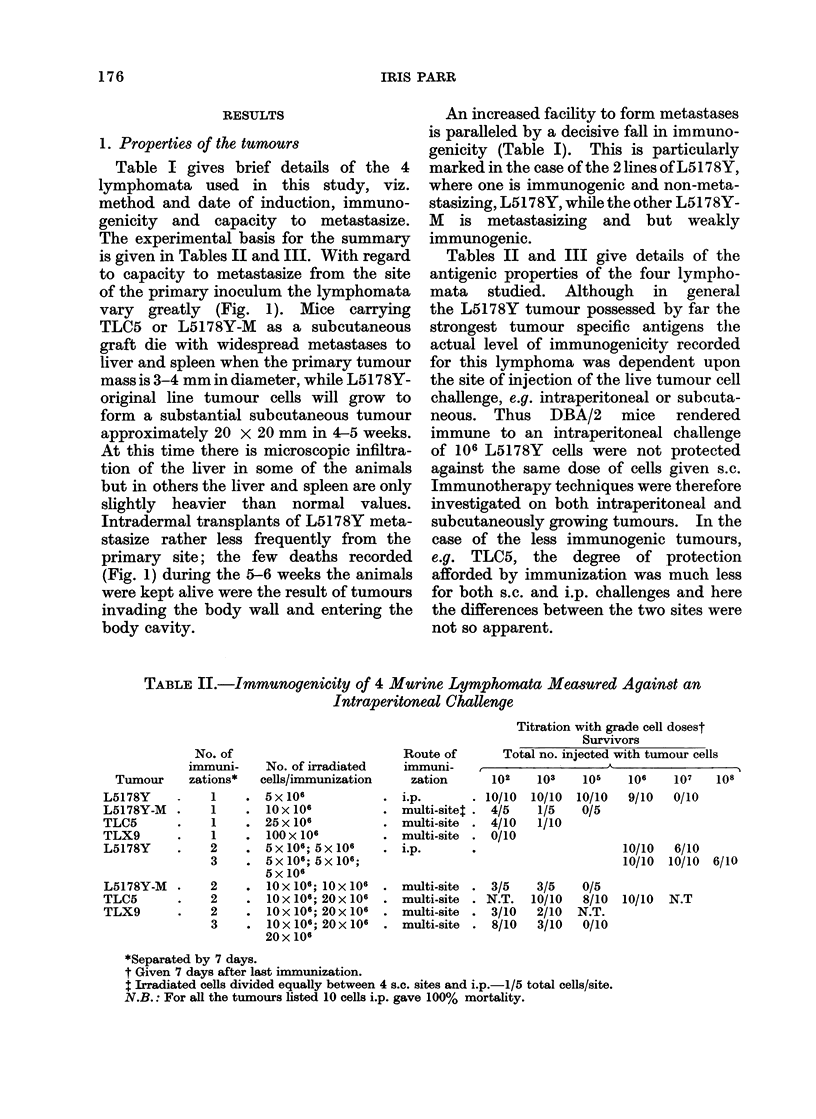

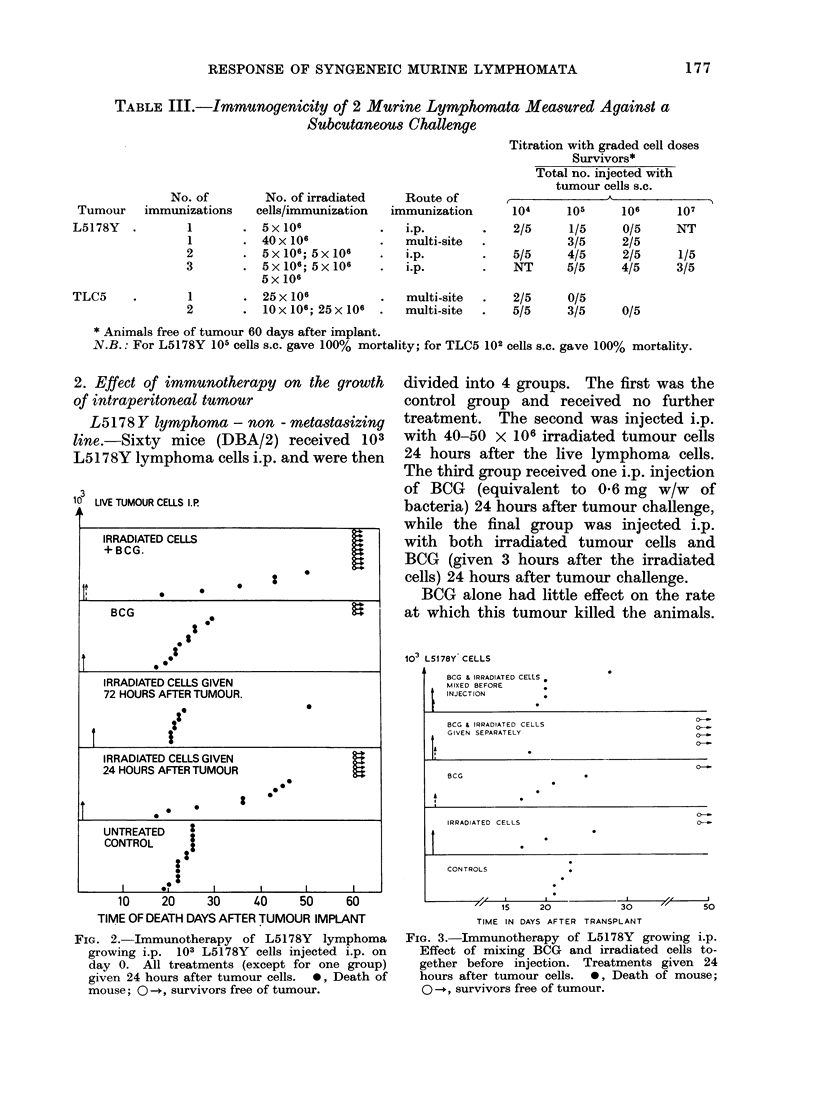

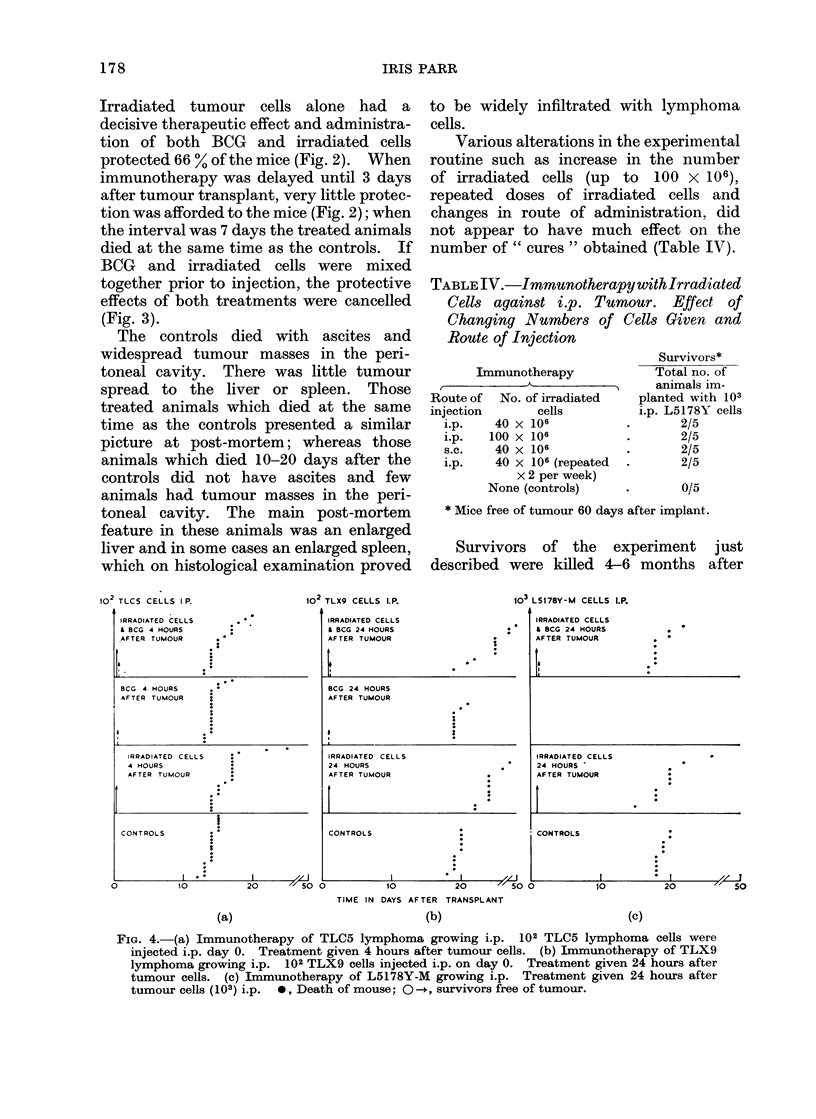

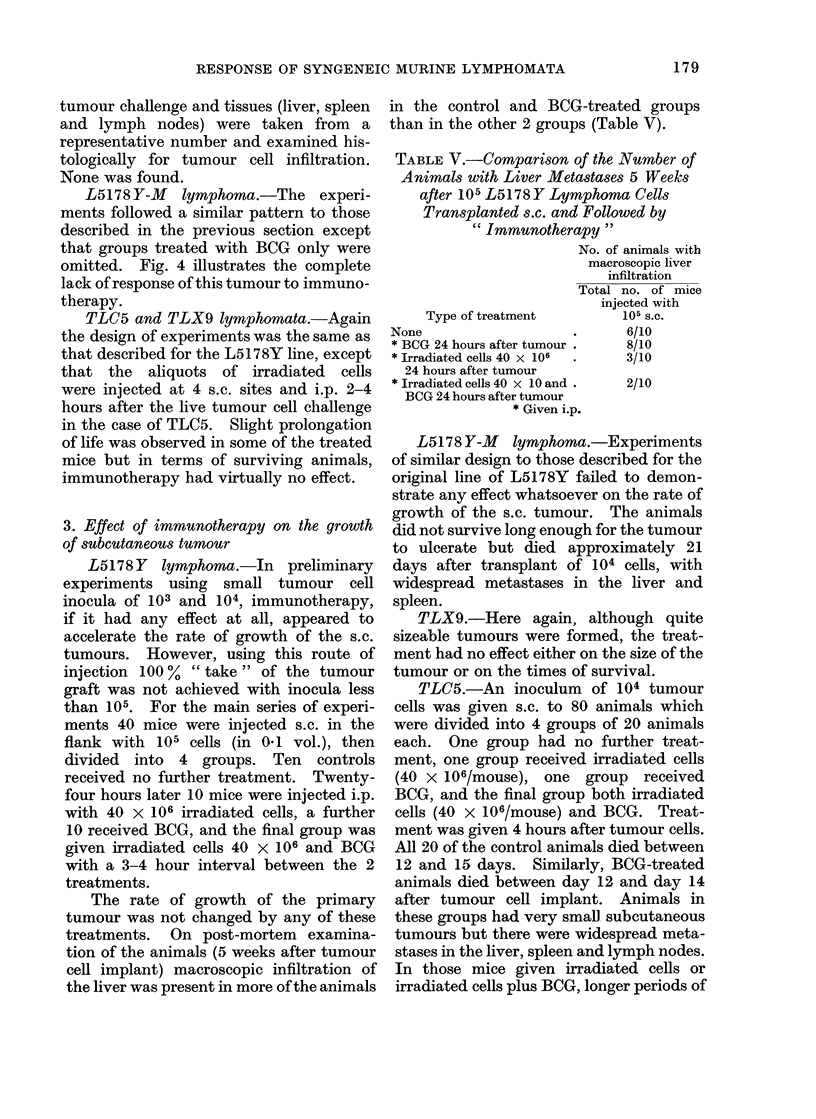

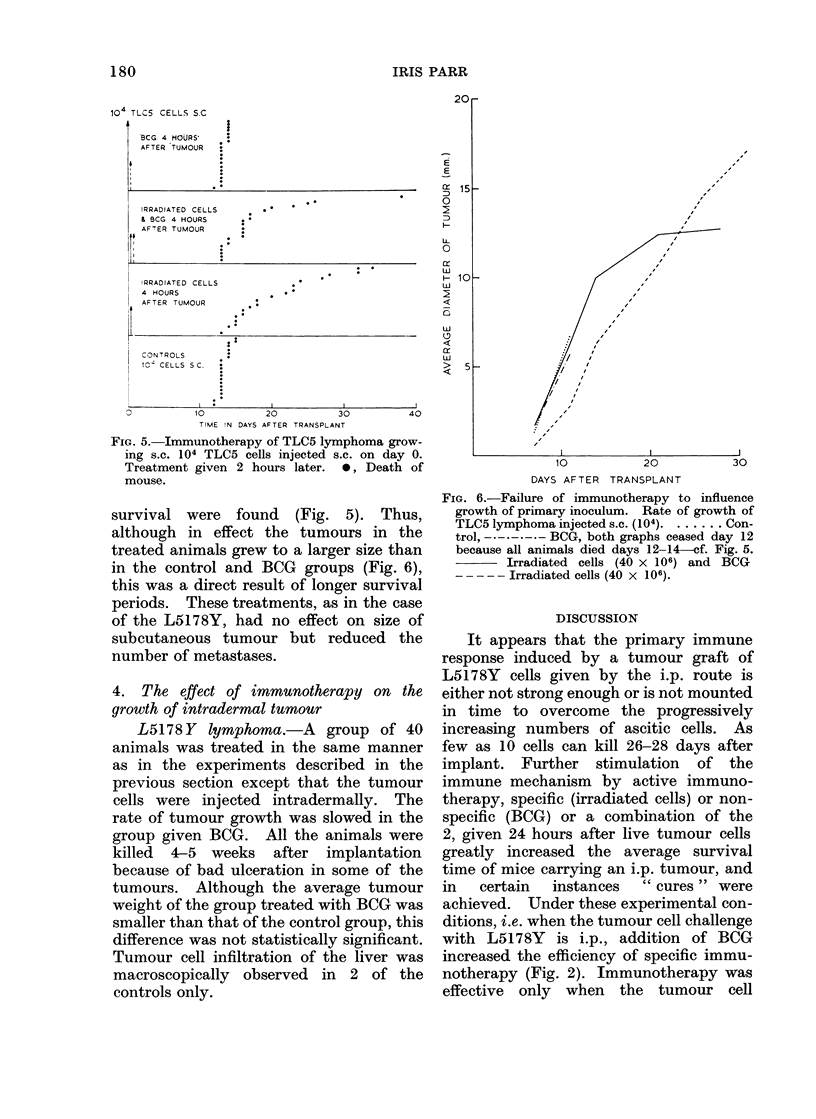

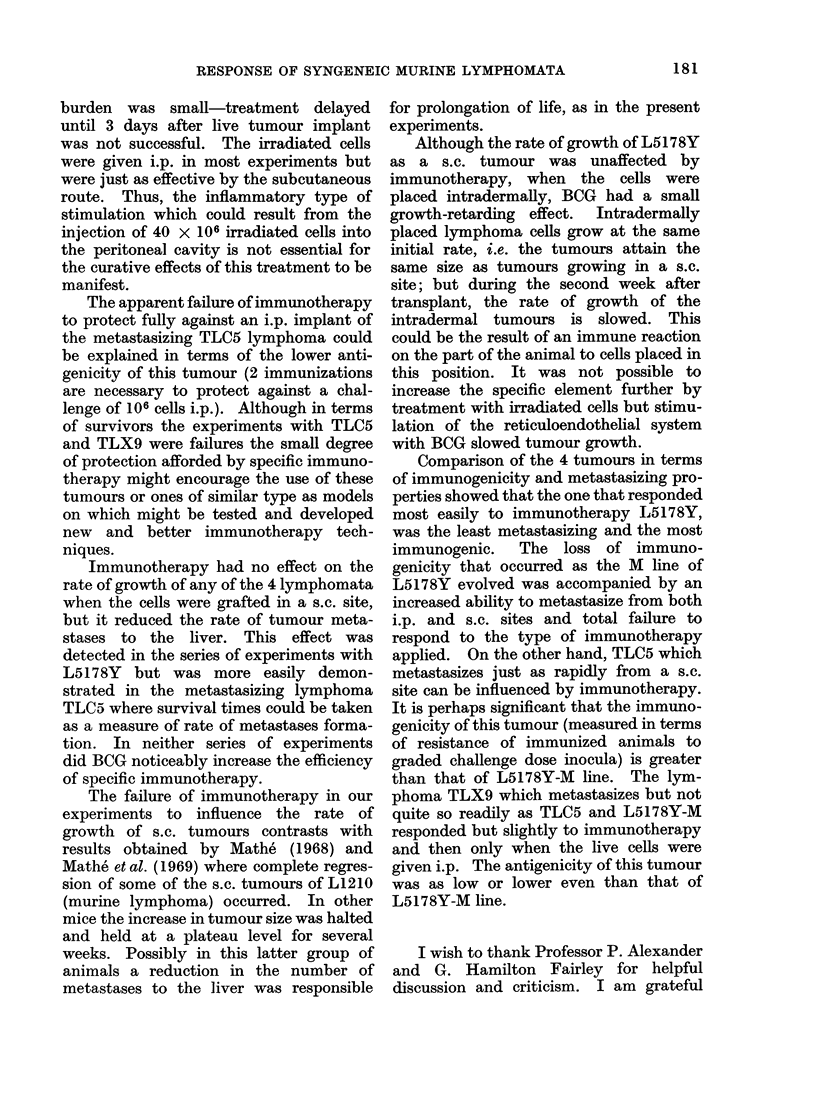

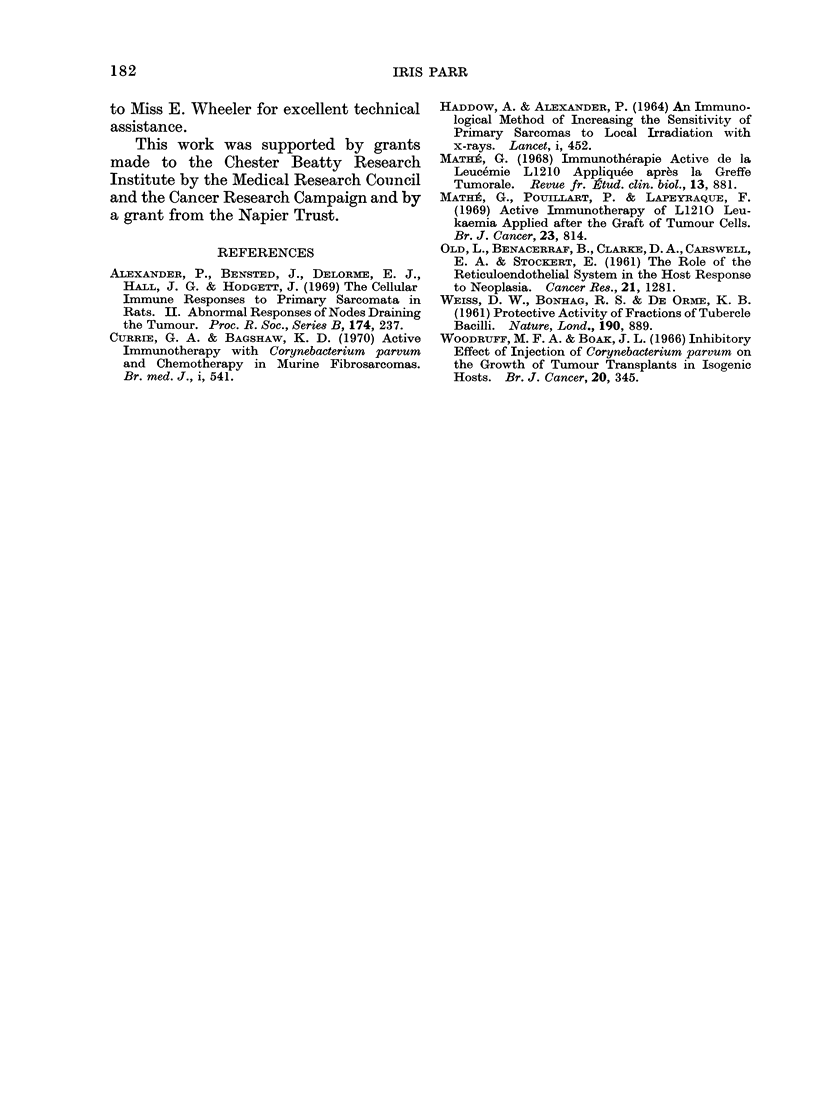


## References

[OCR_01079] Alexander P., Bensted J., Delorme E. J., Hall J. G., Hodgett J. (1969). The cellular immune response to primary sarcomata in rats. II. Abnormal responses of nodes draining the tumour.. Proc R Soc Lond B Biol Sci.

[OCR_01086] Currie G. A. (1970). Active immunotherapy with Corynebacterium parvum and chemotherapy in murine fibrosarcomas.. Br Med J.

[OCR_01092] HADDOW A., ALEXANDER P. (1964). AN IMMUNOLOGICAL METHOD OF INCREASING THE SENSITIVITY OF PRIMARY SARCOMAS TO LOCAL IRRADIATION WITH X RAYS.. Lancet.

[OCR_01103] Mathé G., Pouillart P., Lapeyraque F. (1969). Active immunotherapy of L1210 leukaemia applied after the graft of tumour cells.. Br J Cancer.

[OCR_01120] Woodruff M. F., Boak J. L. (1966). Inhibitory effect of injection of Corynebacterium parvum on the growth of tumour transplants in isogenic hosts.. Br J Cancer.

